# A retrospective analysis of the clinical efficacy in patients treated with *Alternaria alternata* and *Dermatophagoides farinae* immunotherapy

**DOI:** 10.3389/falgy.2024.1453446

**Published:** 2024-08-22

**Authors:** Juan Liu, Jia Yin

**Affiliations:** ^1^ Department of Allergy, Peking Union Medical College Hospital, Chinese Academy of Medical Sciences, Peking Union Medical College, Beijing Key Laboratory of Precision Medicine for Diagnosis and Treatment of Allergic Diseases, Beijing, China; ^2^ National Clinical Research Center for Dermatologic and Immunologic Disease (NCRC-DID), Beijing, China

**Keywords:** efficacy, allergen immunotherapy, *Alternaria alternata*, *Dermatophagoides farinae*, allergic rhinitis, allergic asthma, retrospective

## Abstract

**Background:**

The clinical efficacy of allergen-specific immunotherapy (AIT) for *Alternaria alternata* (*A. alt*) and *Dermatophagoides farinae* (*Der f*) extracts remains largely unknown in China. We sought to retrospectively evaluate the efficacy caused by AIT agents manufactured in China of patients who are sensitized to *A. alt* and *Der f*.

**Methods:**

Patients aged 5–27 years with asthma and perennial allergic rhinitis (AR), and AIT with *A. alt* and *Der f* were recruited, and then classified into two groups: *A. alt*-AIT (*n* = 31) and *A. alt* + *Der f*-AIT group (*n* = 39). All data were gathered retrospectively, including biological parameters, pulmonary function, and symptom and medication scores.

**Results:**

70 patients who underwent *A. alt* and *Der f* AIT were enrolled. A significant improvement was observed in the values of FEV1% (*P* < 0.0001) and MEF 25 (*P* = 0.023) of lung function. Both the rhinitis symptoms and combined symptoms and medication scores for asthma decreased after AIT (by 45.3% and 80.3%, respectively, *P* < 0.0001 for each). Nearly 67% improvement rate (*P* < 0.0001) occurred in rhinoconjunctivitis quality of life, and a great increase existed in Asthma Control Test (ACT) score (*P* < 0.0001) after at least 1 year AIT, although there were no significant changes between these two groups. Besides, no significance was displayed in specific IgE to different allergens.

**Conclusion:**

AIT with *A. alt* and *Der f* extracts had clinical efficacy for many patients in China, with a reduction of symptom and medication scores, and great improvement in spirometry function.

## 1 Introduction

Sensitization to *Alternaria alternata* and *Dermatophagoides farinae* is a common cause of rhinitis and asthma, *Alternaria* is also strongly associated with the persistence and exacerbation of asthma in children (1). Chinese research has reported that *Alternaria alternata* is the most abundant fungal genera in the environment among 667 fungal strains (2). Additionally, the sensitization rate of *Alternaria* reaches 11.9% in several European countries (3), and more than 100 million patients suffer from allergic diseases due to dust mites around the world (4).

In patients with allergen-induced respiratory allergies, AIT is considered effective in preventing the development and progression of new sensitization and is the only approach that maintains long-lasting effects. However, few studies focused on immunotherapy of *Alternaria* allergy (5), and there are few studies evaluating the efficacy of AIT with *Alternaria alternata* extracts in China until now since the clinical efficacy controversy still existed in mold immunotherapy. Besides, numerous studies have confirmed the efficacy of AIT with house dust mite (6), but little is known about the immunotherapy response to *A. alt* and *Der f* extracts in Chinese patients, although a high prevalence of *Alternaria alternata* and *Dermatophagoides farinae* in the grassland of China among pediatrics.

Consequently, We conducted a retrospective study to evaluate clinical efficacy in terms of symptoms, life quality, asthma control, and lung function in patients who underwent AIT with *A. alt* and *Der f* extracts.

## 2 Materials and methods

### 2.1 Study design and patients

All data were collected retrospectively from the outpatient allergy department of Peking Union Medical College Hospital (PUMCH) between January 2022 and January 2023. This study was approved by the IRB of PUMCH (No. S-K1672). All informed consent was completed.

A total of 70 patients aged 5–27 years with a diagnosis of mild or moderate asthma and perennial AR associated with fungal exposure were recruited. All patients presented both positive skin tests (wheal size ≥5 mm) with whole extract of *A. alternata* (PUMCH, Beijing, China) and sIgE (≥0.7 kUA/L) to *Alternaria* (Phadia ImmunoCAP, Uppsala, Sweden). The skin test grading system (1 + to 4+) was used according to our previous study (7). Besides, they all received only *A. alt* extracts AIT or *A. alt* extracts combined with *Der f* extracts AIT for at least 1 year. Subjects were classified into 2 major groups: *A. alt*-AIT group, referred to patients who were individually allergic to *A. alt* and also only accepted AIT with *A. alt* extracts (*n* = 31); *A. alt* + *Der f*-AIT group, referred to patients who were only allergic to *A. alt* + *Der f* and without any other allergens, and subcutaneous injection with *A. alt* + *Der f* extracts at the same time in different arms (*n* = 39). The reasons for the selection of the AIT group of *Alternaria* combined with dust mites are as follows: (1) Both are perennial allergens, seasonal factors will not be considered before the comparison, and further reduce the mixture factors; (2) The two groups of patients are similar in age and are more comparable. Patients who received AIT previously, AIT with pollens or animal dander, suffered from unstable asthma, any nasal disease except for AR, autoimmune or chronic diseases, and incomplete data were excluded from the study.

Demographic and clinical information including sex, eosinophils, T-IgE, spirometry testing, and symptoms and medication scores were acquired.

### 2.2 Specific immunotherapy

Allergen extracts of *A. alt* (PUMCH, China, batch number: S20120006, total protein content 0.5 mg/5 ml) and *Der f* (PUMCH, China, batch number: S20130002, total protein content 1.75 mg/5 ml) were used for AIT with subcutaneous injection following an “up dosing” schedule, and two treatment stages were included. For the initial phase, patients were injected of 0.1 ml as a minimal dose, and increased step by step to achieve the maximum dose of 1.0 ml. The maintenance phase always kept an injection with 1.0 ml until the end of AIT. All the patients sustained an injection twice a week.

### 2.3 Symptom and medication assessment

Symptoms and medication assessment were evaluated by a series of questionnaires that were delivered to patients or their parents. Symptoms related to nose and ocular were recorded using Rhinoconjunctivitis Total Symptom Score (RTSS), including pruritus, nasal congestion, mucus production, sneezing, tearing, and itching. Pulmonary symptoms included cough, wheezing, dyspnea, and exercise-induced asthma (1, no symptoms; 2, mild; 3, moderate; 4, severe). The Rhinoconjunctivitis Quality of Life Questionnaire (RQLQ) and ACT were used to clarify patients' nasal, ocular, and pulmonary control status. Medication score was applied and graded according to Tabar et al. (8) as follows: 0, no rescue medication; 1, topical and/or systemic antihistamines; 2, intranasal corticosteroids; 3, oral corticosteroids.

### 2.4 Statistics analysis

All data were analyzed using Prism 9.0 (GraphPad, California, USA). Descriptive data were performed and presented in tables. Continuous variables were expressed as mean ± SD. The differences between the two groups were analyzed by using an unpaired *t*-test. Within-group comparisons, the paired *t*-test was used. For categorical data, percentages were summarized and the *χ*^2^ test was performed. Statistical significance was set at *P *< 0.05.

## 3 Results

### 3.1 Demographic characteristics

We analyzed and compared the differences in age, gender, IgE titers, pulmonary function, the medication used, another atopic status, and family allergic history between these two groups (Table 1). The mean age of patients was 11.80 ± 3.83 years, and there were far more males than females in both groups. At the baseline, patients in the *A. alt* + *Der f*-AIT group showed higher concentrations of T-IgE (*P* = 0.001) and *Der f*-sIgE (*P* < 0.0001), larger wheals of *Der f*, and increased percentages of eosinophils than the *A. alt*-AIT group (*P* = 0.04). However, there were no statistical differences in respiratory function, medication assessment, and other clinical characteristics between the two treatment groups at the baseline level.

**Table 1:** Demographics and baseline clinical characteristics of subjects.

Variable	Total	*A. alt*-AIT	*A. alt* + *Der f*-AIT	*P’* value
	(*n* = 70)	(*n* = 31)	(*n* = 39)	
Demographic features				
Age (years), mean (SD)	11.80 ± 3.83	12.03 ± 4.32	11.62 ± 3.44	.654^a^
5–11 years, *n* (%)	33 (47%)	13 (42%)	20 (51%)	.437^b^
>12 years, *n* (%)	37 (53%)	18 (58%)	19 (49%)	.437^b^
No. male/female	47/23	20/11	27/12	.677^b^
BMI (kg/m^2^), mean (SD)	17.84 ± 3.52	17.60 ± 3.55	18.03 ± 3.52	.612^a^
Biologic data, mean (SD)				
Total IgE (kU/L)	653.56 ± 689.61	346.07 ± 323.98	897.98 ± 815.42	**.001** ^a^
*A.alt*-sIgE (kUA/L)	27.95 ± 25.54	27.71 ± 25.24	28.14 ± 26.10	.945^a^
*Der f*-sIgE (kUA/L)	11.13 ± 22.46	0.28 ± 0.30	19.68 ± 27.26	**<.0001** ^a^
Wheals class (*A. alt*)	2.25 ± 0.74	2.16 ± 0.58	2.32 ± 0.85	.370^a^
Wheals class (*Der f*)	1.53 ± 1.17	0.68 ± 0.48	2.24 ± 1.09	**<.0001** ^a^
Eos (10^9^/L)	0.40 ± 0.24	0.33 ± 0.20	0.45 ± 0.26	.136^a^
Eos (%)	5.47 ± 3.01	4.43 ± 2.70	6.27 ± 3.05	**.040** ^a^
Respiratory data, mean (SD)				
FEV1 (% pred)	84.46 ± 14.78	84.97 ± 16.32	84.05 ± 13.64	.798^a^
FEV1/FVC (% pred)	94.72 ± 12.08	95.94 ± 9.98	93.76 ± 13.57	.458^a^
PEF (L/min)	84.14 ± 18.99	85.53 ± 16.77	84.62 ± 20.79	.814^a^
MEF 75 (% pred)	70.68 ± 28.01	69.75 ± 25.19	71.41 ± 30.38	.808^a^
MEF 50 (% pred)	70.94 ± 28.47	67.68 ± 25.59	73.53 ± 30.65	.397^a^
MEF 25 (% pred)	63.27 ± 27.79	62.34 ± 28.42	64.02 ± 27.62	.804^a^
Medication data				
ICS (% positive)	62 (89%)	26 (84%)	36 (92%)	.270^b^
LABA (% positive)	39 (56%)	18 (58%)	21 (54%)	.724^b^
LTRA (% positive)	60 (86%)	27 (87%)	33 (85%)	.768^b^
OCS (% positive)	7 (10%)	1 (3%)	6 (15%)	**.092** ^b^
Others				
Eczema, *n* (%)	42 (60%)	16 (52%)	26 (67%)	.202^b^
Pets, *n* (%)	6 (9%)	4 (13%)	2 (5%)	.248^b^
Family allergic history, *n* (%))	38 (54%)	15 (48%)	23 (59%)	.377^b^

AIT, allergen-specific immunotherapy; *A. alt*, *A. alternata*; *Der f*, *Dermatophagoides farinae*; SD, standard deviation; BMI, body mass index; FEV1, forced expiratory volume in the first second; FVC, forced vital capacity; MEF, maximal expiratory flow; PEF, peak expiratory flow; ICS, inhaled corticosteroids; LABA, long-acting *β*_2_ agonist; LTRA, leukotriene receptor agonist; OCS, oral corticosteroids; *P’* value, *A. alt*-AIT vs. *A. alt* + *Der f*-AIT; ^a^*t*-tests; ^b^Chi-square. Bold values signify statistical significance.

### 3.2 *A. alt* or *A. alt* combined with *Der f*-AIT influenced the spirometry function

Spirometry values are displayed in Table 2. In the total of 70 patients, FEV1% of predicted increased after AIT (93.41 ± 8.69) compared to baseline levels (84.46 ± 14.78), these results were similar existed in both the *A. alt*-AIT (*P* = 0.0006) and *A. alt* + *Der f*-AIT group (*P* < 0.0001). In addition, changes in the small airway function (MEF 25) also displayed an upregulation rather in the *A. alt*-AIT patients (*P* = 0.021) or in the *A. alt* + *Der f*-AIT patients (*P* = 0.009), since it was a reliable parameter of respiratory function. Mean PEF increased from 84 to 93 after administering AIT in all patients. However, the values of FEV1/FVC% did not present significant changes between assessments. It was also worth noting that no significance of respiratory function was shown between *A. alt*-AIT and *A. alt* + *Der f*-AIT group at baseline or after treatment.

**Table 2:** Respiratory function of subjects.

Variable	Time points	Total	*A. alt*-AIT	*A. alt* + *Der f*-AIT	*P’* value
		(*n* = 70)	(*n* = 31)	(*n* = 39)	
FEV1 (% pred)	Baseline, mean (SD)	84.46 ± 14.78	84.97 ± 16.32	84.05 ± 13.64	.798
	After AIT	93.41 ± 8.69	93.94 ± 9.72	92.98 ± 7.88	.651
	*P* value	**<.0001**	**.0006**	**<.0001**	
FEV1/FVC (% pred)	Baseline, mean (SD)	94.72 ± 12.08	95.94 ± 9.98	93.76 ± 13.57	.458
	After AIT	96.67 ± 8.88	96.84 ± 9.48	96.53 ± 8.49	.886
	*P* value	.280	.661	.126	
PEF (L/min)	Baseline, mean (SD)	84.14 ± 18.99	85.53 ± 16.77	84.62 ± 20.79	.814
	After AIT	93.20 ± 16.29	92.84 ± 17.04	93.49 ± 15.88	.864
	*P* value	**.003**	**.008**	**.007**	
MEF 75 (% pred)	Baseline, mean (SD)	70.68 ± 28.01	69.75 ± 25.19	71.41 ± 30.38	.808
	After AIT	84.72 ± 19.64	87.15 ± 21.27	82.79 ± 18.30	.360
	*P* value	**.001**	**.001**	**.007**	
MEF 50 (% pred)	Baseline, mean (SD)	70.94 ± 28.47	67.68 ± 25.59	73.53 ± 30.65	.397
	After AIT	78.76 ± 21.43	79.26 ± 23.55	78.36 ± 19.89	.863
	*P* value	.069	**.008**	.237	
MEF 25 (% pred)	Baseline, mean (SD)	63.27 ± 27.79	62.34 ± 28.42	64.02 ± 27.62	.804
	After AIT	73.73 ± 26.10	73.67 ± 27.67	74.93 ± 24.42	.987
	*P* value	**.023**	**.021**	**.009**	

AIT, allergen-specific immunotherapy; *A. alt*, *A. alternata*; *Der f*, Dermatophagoides farinae; SD, standard deviation; FEV1, forced expiratory volume in the first second; FVC, forced vital capacity; MEF, maximal expiratory flow; PEF, peak expiratory flow; *P’* value, *A. alt*-AIT vs. *A. alt* + *Der f*-AIT. Bold values signify statistical significance.

Besides, we also analyzed the changes in FEV1% levels of individual patients. Patients who had an FEV1% value lower than 70% before immunotherapy, showed not much improvement after AIT treatment, which only recovered to about 80%–90% of predicted FEV1%, whether in *A. alt*-AIT or *A. alt* + *Der f*-AIT patients.

### 3.3 Changes in biologic parameters after *A. alt* or *A. alt* combined with *Der f*-AIT

In the *A. alt*-AIT group, the level of T-IgE demonstrated a higher tendency after AIT (*P* = 0.005) while there were no significant differences in the *A. alt* + *Der f*-AIT group (Table 3). However, the changes that occurred similarly before AIT, and T-IgE values still showed nearly double the level in the patients who accepted *A. alt* and *Der f* mixture extracts than in the *A. alt*-AIT patients (*P* = 0.015). Besides, specific IgE to *A. alt* and the numbers of eosinophils with no obvious difference between before and after AIT, and also without significance between group-analysis. However, the *Der f*-sIgE titers reached a high concentration in the *A. alt* + *Der f*-AIT group all the time. Meanwhile, the proportions of eosinophils reduced from 6.27 to 4.39 after the *A. alt* combined with *Der f* immunotherapy compared with the baseline (*P* = 0.038).

**Table 3:** Biologic data of subjects.

Variable	Time points	Total	*A. alt*-AIT	*A. alt* + *Der f*-AIT	*P’* value
		(*n* = 70)	(*n* = 31)	(*n* = 39)	
T-IgE (KU/L)	Baseline, mean (SD)	653.56 ± 689.61	346.07 ± 323.98	897.98 ± 815.42	.**001**
	After AIT	659.22 ± 773.40	457.72 ± 412.40	888.97 ± 935.73	.**015**
	*P* value	.458	**.005**	.423	
*A. alt*-sIgE (kUA/L)	Baseline, mean (SD)	27.95 ± 25.54	27.71 ± 25.24	28.14 ± 26.10	.945
	After AIT	22.34 ± 20.17	24.22 ± 21.73	20.76 ± 18.91	.917
	*P* value	.417	.329	.170	
*Der f* -sIgE (kUA/L)	Baseline, mean (SD)	11.13 ± 22.46	0.28 ± 0.30	19.68 ± 27.26	**<.0001**
	After AIT	9.42 ± 17.89	0.29 ± 0.27	12.84 ± 19.98	.**015**
	*P* value	.552	.308	.487	
Eos (10^9^/L)	Baseline, mean (SD)	0.40 ± 0.24	0.33 ± 0.20	0.45 ± 0.26	.136
	After AIT	0.33 ± 0.27	0.34 ± 0.35	0.32 ± 0.19	.788
	*P* value	.243	.580	.075	
Eos (%)	Baseline, mean (SD)	5.47 ± 3.01	4.43 ± 2.70	6.27 ± 3.05	.**040**
	After AIT	4.24 ± 2.42	4.08 ± 2.88	4.39 ± 1.99	.682
	*P* value	**.046**	.995	**.038**	

AIT, allergen-specific immunotherapy; *A. alt*, *A. alternata*; *Der f*, *Dermatophagoides farinae*; SD, standard deviation; Eos, eosinophils; *P’* value, *A. alt*-AIT vs. *A. alt* + *Der f*-AIT. Bold values signify statistical significance.

### 3.4 Symptom and medication scores

Symptom and medication scores in patients according to the condition were recorded in Table 4. For all patients with AR and asthma, there was a greater improvement (45.3%) in RTSS after immunotherapy (1.37 ± 0.52) than baseline (1.99 ± 0.50), as well as the better RQLQ acquired when patients administrated AIT at least one year (improvement rate: 66.9%, 2.62 ± 0.92 vs. 1.57 ± 0.56). Meanwhile, a significant reduction was observed in the RMS at the baseline level relative to the immunotherapy level (8.57 ± 6.89 vs. 4.11 ± 7.38, *P* = 0.0003). Differences in the CSMS and ACT scores between baseline and at least one year of AIT were statistically significant (*P* < 0.0001 in both cases). When analyzing the different allergen extracts of AIT separately, we found better improvement in all symptom and medication scores in the *A. alt*-AIT population, rather than patients in the *A. alt* + *Der f*-AIT group. However, we did not find the differences that existed in *A. alt*-AIT and *A. alt* + *Der f*-AIT groups whether before or after immunotherapy.

**Table 4:** Symptom and medication data of subjects.

Variable	Time points	Total	*A. alt*-AIT	*A. alt* + *Der f*-AIT	*P’* value
		(*n* = 70)	(*n* = 31)	(*n* = 39)	
RTSS	Baseline, mean (SD)	1.99 ± 0.50	1.95 ± 0.51	2.01 ± 0.50	.625
	After AIT	1.37 ± 0.52	1.25 ± 0.36	1.46 ± 0.61	.104
	*P* value	**<.0001**	**.0001**	.423	
RQRL	Baseline, mean (SD)	2.62 ± 0.92	2.54 ± 0.97	2.68 ± 0.89	.523
	After AIT	1.57 ± 0.56	1.47 ± 0.40	1.66 ± 0.66	.153
	*P* value	**<.0001**	**<.0001**	**<.0001**	
RMS	Baseline, mean (SD)	8.57 ± 6.89	7.87 ± 5.22	9.13 ± 7.99	.452
	After AIT	4.11 ± 7.38	2.79 ± 4.54	5.15 ± 8.95	.185
	*P* value	**.0003**	**<.0001**	**.0001**	
CSMS	Baseline, mean (SD)	6.78 ± 2.80	6.29 ± 1.97	7.18 ± 3.29	.189
	After AIT	3.76 ± 2.95	3.44 ± 2.68	4.01 ± 3.15	.426
	*P* value	**<.0001**	**<.0001**	**<.0001**	
ACT	Baseline, mean (SD)	20.21 ± 3.46	19.94 ± 3.40	20.44 ± 3.53	.551
	After AIT	24.21 ± 1.21	24.03 ± 1.28	24.36 ± 1.16	.267
	*P* value	**<.0001**	**<.0001**	**<.0001**	

AIT, allergen-specific immunotherapy; *A. alt*, *A. alternata*; *Der f*, *Dermatophagoides farinae*; SD, standard deviation; RTSS, rhinoconjunctivitis total symptom score; RQRL, rhinoconjunctivitis quality of life questionnaire; RMS, rhinoconjunctivitis medication score; CSMS, combined symptom and medication score; ACT, asthma control test; *P’* value, *A. alt*-AIT vs. *A. alt* + *Der f*-AIT. Bold values signify statistical significance.

**Figure 1:**
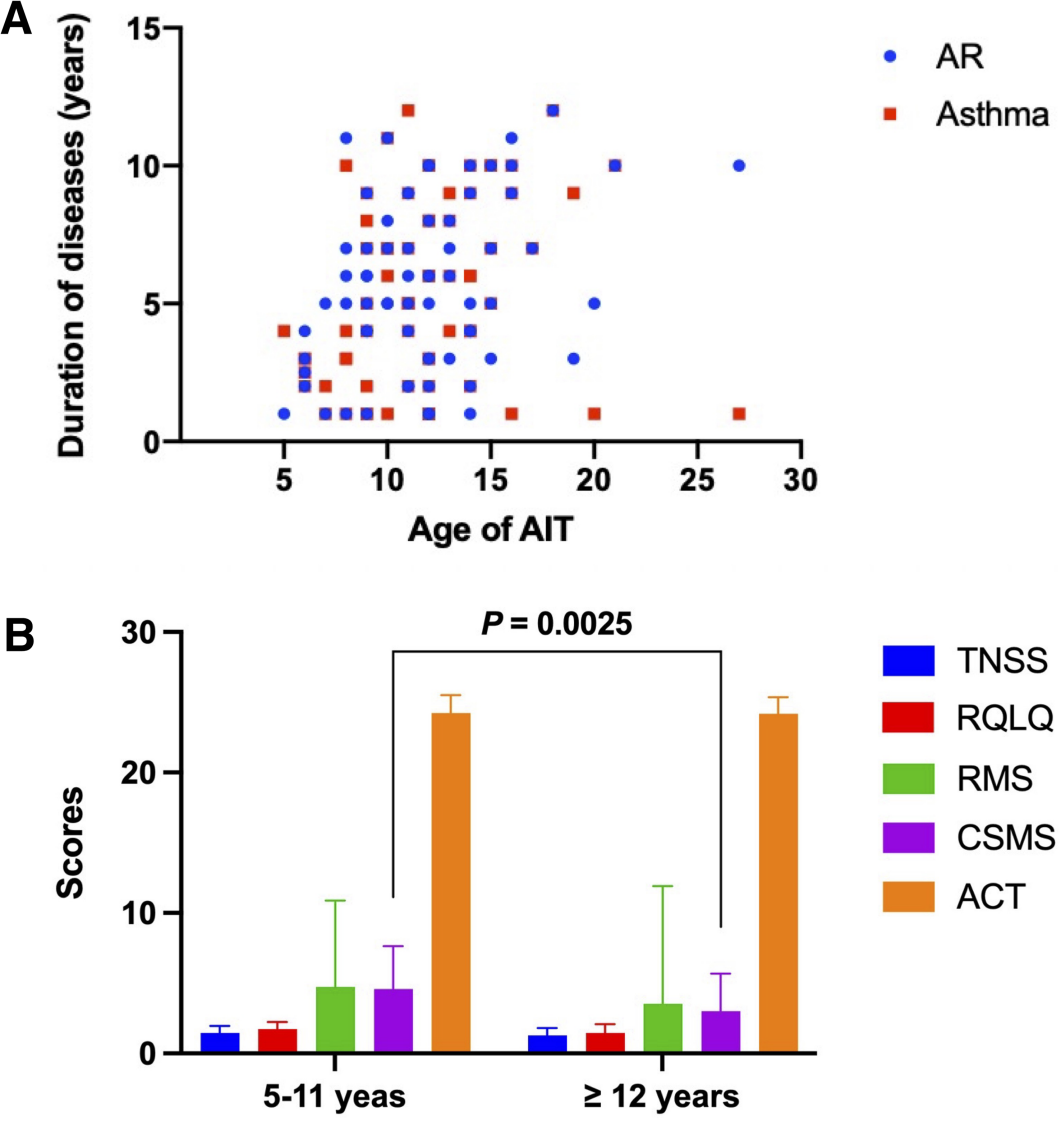
The differences in administrating AIT and scoring symptoms and medication according to the age of all patients. **(A)** The age of patients who received AIT. **(B)** Analysis of symptom and medication scores in two different age groups. AIT, allergen-specific immunotherapy.

We further analyzed the differences in symptom and medication scores according to the age of all patients (Figure 1**)**. The age of patients receiving AIT focused on 5–15 years old, and the course of AR combined with asthma ranged from 5 to 10 years (Figure 1A). After classifying the age into two different scopes (5–11 years vs. ≥12 years) (Figure 1B), results demonstrated that a statistical significance only occurred in the evaluation of CSMS. Patients in 5–11 years showed a better response in CSMS compared to the older after immunotherapy (*P* = 0.0025).

**Figure 2:**
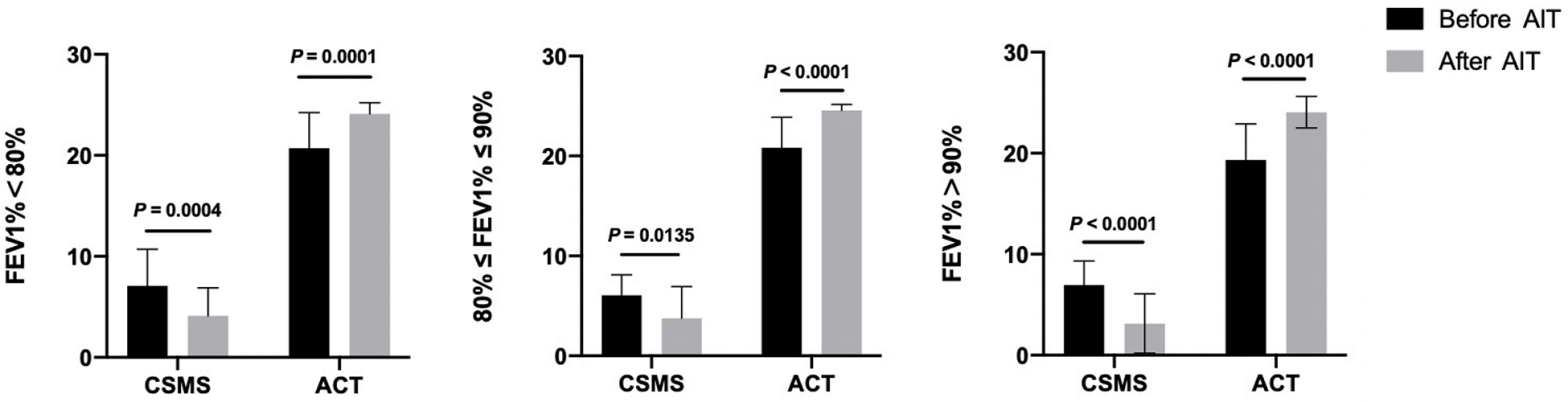
The improvement of CSMS and ACT in different FEV1% grades. CSMS, combined symptom and medication score; ACT, Asthma Control Test; FEV1%, forced expiratory volume in the first second.

When analyzing the improvement of CSMS and ACT in different FEV1% grades separately (Table 5, Figure 2), we classified patients into three subgroups according to the values of FEV1%. Results displayed that patients in the FEV1% >90% group (*n* = 27) had the greatest response of CSMS (*P* < 0.0001) and ACT (*P* < 0.0001) among the three groups. Followed by the group of FEV1% <80% (*n* = 25), which also showed a 72% reduction in the mean CSMS (*P* = 0.0004) and a significant elevation in ACT (*P* = 0.0001). Although the group of 80% ≤FEV1% ≤90% (*n* = 18) shared similar improvements in these two scores, CSMS only changed 61.4% (*P* = 0.014).

**Table 5:** Classification of FEV1% in all subjects.

Variable	Time points	FEV1% < 80%	80% ≤ FEV1% ≤ 90%	FEV1% > 90%
		(*n* = 25)	(*n* = 18)	(*n* = 27)
CSMS	Baseline, mean (SD)	7.09 ± 3.61	6.07 ± 2.05	6.97 ± 2.36
	After AIT	4.12 ± 2.76	3.76 ± 3.18	3.15 ± 2.93
	*P* value	**.0004**	**.014**	**<.0001**
ACT	Baseline, mean (SD)	20.72 ± 3.52	20.83 ± 3.05	19.33 ± 3.58
	After AIT	24.12 ± 1.09	24.56 ± 0.62	24.07 ± 1.57
	*P* value	**.0001**	**<.0001**	**<.0001**

FEV1, forced expiratory volume in the first second; AIT, allergen-specific immunotherapy; SD, standard deviation; CSMS, combined symptom and medication score; ACT, asthma control test. Bold values signify statistical significance.

To determine whether individuals receiving inhaled corticosteroids before immunotherapy had different degrees of change in symptom and medication scores after AIT, patients were initially grouped into those who had never been given ICS (non-ICS, *n* = 8), those who had previously been on low (low-ICS, *n* = 43), middle (middle-ICS, *n* = 15), or high ICS (high-ICS, *n* = 4) therapy (Table 6). Our results showed that CSMS and ACT scores were comparable between baseline and receiving AIT at least 1 year in non-ICS patients. In comparison, followed by AIT, low ICS therapy in individuals presented the most statistical significance in CSMS and ACT improvement (*P* < 0.0001). Individuals in the middle ICS-taken group revealed a reduction of 58.8% in CSMS evaluation (*P* = 0.004), and also a significant difference between before and after AIT was observed for middle ICS treatment in the ACT (*P* = 0.0001). In addition, this same reduction trend of CSMS occurred in the high ICS treatment patients (*P* = 0.035), although only a few patients inhaled high doses of corticosteroids. For further comparison of ACT scores in this group, the difference between before and after AIT was not significant.

**Table 6:** Classification of ICS used in all subjects.

Variable	Time points	Non-ICS	Low-ICS	Middle-ICS	High-ICS
		(*n* = 8)	(*n* = 43)	(*n* = 15)	(*n* = 4)
CSMS	Baseline, mean (SD)	3.67 ± 0.89	6.24 ± 1.68	8.72 ± 3.33	11.56 ± 2.88
	After AIT	2.63 ± 2.52	3.25 ± 2.67	5.49 ± 2.63	4.97 ± 5.37
	*P* value	.318	**<.0001**	**.004**	**.035**
ACT	Baseline, mean (SD)	22.13 ± 3.60	19.79 ± 3.54	20.47 ± 2.72	19.75 ± 4.99
	After AIT	25.00 ± 0.00	23.98 ± 1.44	24.33 ± 0.72	24.50 ± 0.58
	*P* value	.059	**<.0001**	**.0001**	.161

ICS, inhaled corticosteroids; AIT, allergen-specific immunotherapy; SD, standard deviation; CSMS, combined symptom and medication score; ACT, asthma control test. Bold values signify statistical significance.

## 4 Discussion

Few studies focused on the efficacy of AIT with fungal extracts worldwide, especially for *Alternaria alternata* (9–11), although AIT has been concluded effective for mold allergy by the American Academy of Allergy, Asthma & Immunology (12). More importantly, little research on immunotherapy with *A. alt* and *Der f* extracts has been published in China, except for a real-world analysis of a small sample published by us (13). Here, we retrospectively studied the efficacy of *A. alternata* and *Dermatophagoides farinae* which was produced by PUMCH in the course of clinical usage. Results in our study displayed that perennial AIT for *A. alt* and *Der f* were significantly improving the symptom and medication scores for AR and asthma, along with the quality of life, and asthma control, and had a large increase in respiratory function.

In our data analysis, the mean age of the patients was nearly 12 years old and the proportion of males was higher than females. This young age reflects that sensitization to mold and *Der f* had higher incidence at early ages (14, 15), as well as the limitation of inclusion criteria only to *A. alt* immunotherapy and *A. alt* combined with *Der f* immunotherapy. A higher prevalence of *A. alternata* sensitization related to males was confirmed by several studies (16, 17). Furthermore, we did not find a significance of a family allergic history and pets in both groups at the baseline level, since the progress of asthma was influenced by genetic and environmental factors (18).

A significant increase in pulmonary function was demonstrated as a result of AIT with both *Alternaria alternata* and *Dermatophagoides farinae* extracts. A study performed by Fielding et al. (19) showed that falling FEV1% might be related to the exacerbation of asthma and the change in FEV1% can be a valuable predictor for the risk of asthma. Therefore, these results demonstrated the important role of FEV1% in asthma assessment. However, many immunotherapy studies of *A. alternata* and *Dermatophagoides farinae* did not evaluate the response of pulmonary, because they mainly highlight the role of symptom and medication scores. Our study concluded a remarkable improvement of FEV1%, PEF, and MEF 25 after accepting immunotherapy, which is similar to the results of Kim et al. (20). It is also worth noting that the lower the value of FEV1% sustained before immunotherapy, the harder to recover to a higher level followed by AIT, although we did not know the reasons. Nevertheless, the variations of respiratory function remained similar change between *A. alt*-AIT and *A. alt* + *Der f*-AIT patients, which indicated the improvement of lung function after immunotherapy was not related to the type of allergen, but the mechanisms also might be explored further.

Regarding the biological parameters, including IgE and eosinophils in serum, we did not find prominent variations in both groups either before or after immunotherapy. Studies have proved that changes in IgE values are probably not a vital mechanism of the AIT (21), and the study conducted by Uriarte et al. (22) also did not report remarkable variations at one year of AIT. Additionally, a reduction of sIgE levels might be observed after long AIT over several years (23). Patients in the *A. alt* combined *Der f* immunotherapy group presented a higher level of T-IgE may be attributed to the sensitization to *Der f* excepted for *A. alternata*. Researchers found decreases in nasal and sputum eosinophilia predicted the improvement of asthma control (22). Meanwhile, The activation and recruitment of eosinophils to target organs could be reduced during AIT (24). This same result can be found in our study.

The clinical improvements were significantly followed by at least one year of AIT in our study analysis, these improvements included RTSS, RMS, RQLQ, CSMS, and ACT. The majority of clinical trials of *A. alternata* have emphasized and confirmed a significant improvement in symptom and medication scores compared with placebo (8, 10). However, there was no clinical data shown in China, although both of the extracts had been administrated in patients for a long history. Our study displayed that both nasal and respiratory symptoms largely decreased after AIT, and we found no association between clinical efficacy and a unique pattern of sensitization to different allergens, which is similar to the study of cat and dog immunotherapy (22). Furthermore, our findings about the symptom and medication scores in different ages of all patients were interesting, younger patients indicated better response to CSMS than the older, two possible reasons might explain it. Firstly, the earlier immunotherapy for asthma patients, the better the therapeutic effect acquired. Secondly, younger children were mainly supervised by their parents and showed good compliance. At the same time, our results for the relationship between diverse FEV1% grades and CSMS and ACT were meaningful. The higher predicted values of FEV1% among patients, the probably greater improvement of CSMS and ACT scores were obtained, which suggested that patients with AR combined with asthma could administrate immunotherapy as soon as possible without damage to lung function. More importantly, most of the patients still inhaled corticosteroids during AIT, we found patients with a low dose of ICS before AIT acquired the best improvement in CSMS and ACT. This finding may be due to the mild symptoms of these patients relative to other middle or high-ICS subjects. In overall terms, the parameters we applied to estimate clinical efficacy suggested that immunotherapy with *Alternaria alternata* and *Dermatophagoides farinae* extracts produced in China were beneficial to AR and asthma patients.

A lack of safety assessment was the major limitation in our study since AIT shows a risk of local and systemic side effects. Another issue was the lack of placebo control in immunotherapy, we only compared clinical efficacy before and after AIT, and a further prospective study is required to perform with a large cohort. Detecting the variations of the fractional exhaled nitric oxide (FeNO) value in all patients through immunotherapy is significant since it is recommended by the international guideline for monitoring asthma (25). Furthermore, we did not record the change in wheel size after immunotherapy because of the partial lack of data.

## 5 Conclusion

Our results proved that immunotherapy with *Alternaria alternata* and *Dermatophagoides farinae* extracts could significantly improve the pulmonary function, and symptom and medication scores of asthma patients. There was no difference between the immunotherapy of *Der f* combined with *A. alt* and the immunotherapy of *A. alt* alone. In addition, we evaluated the improvement of patients' symptoms and medication by grading different age groups, pulmonary function, and the use of ICS, which was of clinical significance. Therefore, AIT with *A. alt* and *Der f* extracts showed great clinical efficacy for patients in China.

## Data Availability

The original contributions presented in the study are included in the article/Supplementary Material, further inquiries can be directed to the corresponding author.
